# Nursing Leadership—Mapping the Challenges of Newly Qualified Nurses in Hospital Units: A Scoping Review Protocol

**DOI:** 10.3390/nursrep15060215

**Published:** 2025-06-12

**Authors:** Maria Aurélia da Silveira Assoni, Ana Lucia do Nascimento Braga, Viviane Nunes Tosta da Cunha, Jaquelini Brito Francisco, Guilherme Grici Hisatomi, William Donegá Martinez, Emerson Roberto dos Santos, João Daniel de Souza Menezes, Matheus Querino da Silva, Alex Bertolazzo Quitério, Janaína Aparecida de Sales Floriano, Rauer Ferreira Franco, Andressa Karina Stefani, Eliana Fazuoli Chubaci, Soraya Palazzo, Flávia Cristina Custódio, Daniela Gonçalves Faustino, Helena Landim Gonçalves Cristóvão, Ana Beatriz Proni Câmara, Martins Fideles dos Santos Neto, Denise Cristina Móz Vaz Oliani, Cristina Prata Amendola, Neuza Alves Bonifácio, Luís Cesar Fava Spessoto, Nádia Antônia Aparecida Poletti, Maysa Alahmar Bianchin, Josimerci Ittavo Lamana Faria, Alba Regina de Abreu Lima, Vânia Maria Sabadoto Brienze, Júlio César André

**Affiliations:** 1 Center for Studies and Development of Health Education—CEDES, FAMERP—Faculty of Medicine of São José do Rio Preto, São José do Rio Preto 15090-000, Brazil; maria.assoni@prof.saocamilo-sp.br (M.A.d.S.A.); enfabraga@hotmail.com (A.L.d.N.B.); jaquelinienf@gmail.com (J.B.F.); william.martinez@edu.famerp.br (W.D.M.); emerson.santos@edu.famerp.br (E.R.d.S.); matheus.querino@edu.famerp.br (M.Q.d.S.); alex.quiterio@edu.famerp.br (A.B.Q.); janaina.floriano@edu.famerp.br (J.A.d.S.F.); andressaartigos@bol.com.br (A.K.S.); li_chubaci@hotmail.com (E.F.C.); helena.cristovao@edu.famerp.br (H.L.G.C.); ana.camara@edu.famerp.br (A.B.P.C.); maysa@famerp.br (M.A.B.); josimerci.faria@edu.famerp.br (J.I.L.F.); alba.lima09@gmail.com (A.R.d.A.L.); vania.brienze@hospitaldebase.com.br (V.M.S.B.); julio.andre@edu.famerp.br (J.C.A.); 2 Santa Casa de Misericórdia de Barretos, Pio XII Foundation, Barretos 14780-320, Brazil; viviane.tosta@santacasabarretos.com.br; 3 Bebedouro Regional Hospital, Pio XII Foundation, Barretos 14784-370, Brazil; toshybas@yahoo.com; 4 Brazil University, Fernandópolis 15613-899, Brazil; rauer.franco@edu.famerp.br; 5 São Camilo University Center, São Paulo 04263-200, Brazil; enfcentrocirurgico@saocamilo-sp.br; 6 UNIFUNEC—University Center of Santa Sé do Sul, Santa Fé do Sul 15775-000, Brazil; fccustodio@funecsantafe.edu.br; 7 FUNFARME—Hospital de Base, São José do Rio Preto 15090-000, Brazil; daniela.faustino@hospitaldebase.com.br; 8 NSIB—National Cancer Institute (INCA), Rio de Janeiro 20230-130, Brazil; martins.neto@ensino.inca.gov.br; 9 University Hospital Center Cova da Beira, University of Beira Interior, 6200-251 Covilhã, Portugal; vaz.oliani@gmail.com; 10 Pio XII Foundation—Hospital de Amor de Barretos, Barretos 14784-390, Brazil; diretoriamedica@hospitaldeamor.com.br; 11 Faculty of Veterinary Medicine, Universidade Estadual Paulista Júlio de Mesquita Filho, Araçatuba 16050-680, Brazil; neuzabonifacio@gmail.com; 12 Institute of Health Sciences, Paulista University, Araçatuba 16018-555, Brazil; 13 FAMERP—Faculty of Medicine of São José do Rio Preto, São José do Rio Preto 15090-000, Brazil; lcspessoto@gmail.com (L.C.F.S.); nadia@famerp.br (N.A.A.P.)

**Keywords:** graduate nurse, workplace preparation, incivility, leadership, newly qualified nurses, organisational behaviour

## Abstract

**Background/Objectives**: Leadership in nursing plays a pivotal role in the provision of high-quality healthcare. It is, therefore, of paramount importance to understand the challenges encountered by newly qualified nurses when assuming leadership responsibilities. This scoping review protocol aims to identify and map these challenges, thereby facilitating the development of more effective interventions and support programmes. The data collection and analysis were conducted from January 2024 to March 2024. **Methods**: The review was conducted in accordance with the Joanna Briggs Institute (JBI) guidelines for scoping reviews and the Preferred Reporting Items for Systematic reviews and Meta-Analyses extension for Scoping Reviews (PRISMA-ScR) recommendations. A comprehensive search was undertaken across MEDLINE, CINAHL, Web of Science, Scopus, LILACS, and SciELO databases, in addition to a search for the grey literature. Study selection was performed by two independent reviewers, applying pre-defined inclusion and exclusion criteria. The data were extracted using a standardised form and analysed descriptively. **Results**: The review identified several key challenges faced by newly qualified nurses in leadership roles, including conflict management, decision-making under pressure, and effective communication. These findings highlight the complexity of transitioning from academic training to professional practice. The results provide a comprehensive overview of these challenges, enabling the identification of gaps in the existing knowledge base and informing the direction of future research and interventions. **Conclusions**: The findings of this review will contribute to the enhancement of healthcare quality and the promotion of the professional development of newly qualified nurses. This research underscores the importance of developing targeted interventions and support systems to address the specific challenges identified, thereby influencing nursing practice and policy.

## 1. Introduction

Leadership in nursing is of critical importance to the delivery of high-quality healthcare, exerting a direct influence on team performance and patient outcomes [1,2,3]. However, newly qualified nurses frequently encounter significant challenges when transitioning into leadership positions. The hospital environment, characterised by its inherent complexity, high stakes, and dynamic nature, presents a particularly challenging context for this transition, necessitating a specific focus on the experiences within these units. This is attributable to the inherent complexity of the hospital environment and the necessity of integrating technical proficiencies with managerial and interpersonal competencies [4,5,6,7,8]. These challenges may encompass the management of conflict, including interpersonal, intrapersonal, and organisational conflicts, decision-making under conditions of duress, effective communication with team members, and adaptation to the multifaceted expectations of a leadership role [9,10]. Furthermore, newly qualified nurses in leadership roles may face unique pressures stemming from external influences, such as controlling family members or other stakeholders, particularly when dealing with patients lacking capacity.

The transition from academic training to professional practice necessitates that newly qualified nurses apply theoretical knowledge to real-world scenarios, which are often characterised by complexity and unpredictability. A paucity of practical experience, coupled with the need to cultivate leadership acumen, can engender insecurity and difficulties in the effective management of the nursing team [4,11,12]. Furthermore, effective leadership mandates the capacity to foster a collaborative and safe working environment, wherein team members feel valued and motivated to pursue shared objectives [13,14].

The extant scientific literature has demonstrated that the development of leadership competencies in newly qualified nurses is essential for ensuring the provision of high-quality healthcare and fostering professional satisfaction [15,16,17]. Mentorship programmes, training initiatives in communication and conflict management skills, and the provision of support from experienced leaders can contribute to the professional success of these individuals in their leadership functions [11,18,19,20]. Nevertheless, a lacuna persists in our understanding of the specific challenges confronted by newly qualified nurses in leading nursing teams within the context of hospital units [13,15,18]. While existing studies address aspects of new graduate transition and nursing leadership more broadly, this scoping review protocol is specifically designed to systematically identify and comprehensively map the salient and specific challenges faced by newly qualified nurses (defined as up to two years post-graduation experience) exclusively within the unique environment of hospital units. This focused approach aims to provide a granular understanding of the challenges pertinent to this critical setting and population, thereby distinguishing it from broader reviews and laying a robust foundation for targeted interventions.

Considering the context, the present scoping review protocol has the overarching aim of identifying and mapping the principal challenges faced by newly qualified nurses in leading nursing teams in hospital units. This is with a view to providing a robust foundation for the development of targeted interventions and support programmes that will promote the success of these professionals in their leadership endeavours.

## 2. Materials and Methods

This scoping review adheres to the Joanna Briggs Institute (JBI) guidelines for scoping reviews [21] and was conducted in accordance with the recommendations of the Preferred Reporting Items for Systematic reviews and Meta-Analyses extension for Scoping Reviews (PRISMA-ScR) [22]. To ensure transparency and preclude duplication of effort, the review protocol has been registered with the Open Science Framework (OSF): DOI 10.17605/OSF.IO/U8N7P.

### 2.1. Study Design

This study adopts a scoping review design, with the aim of identifying and mapping the primary challenges encountered by newly qualified nurses in leading nursing teams within hospital units. The scoping review methodology is apposite for this topic, as it seeks to delineate the extent and nature of the available evidence, rather than evaluating the quality or effect of specific interventions [23]. This scoping review is guided by the five stages proposed by Arksey and O’Malley (2005) [23]:

- Identifying the research question: The central research question is precisely articulated.
- Identifying relevant studies: A systematic search of relevant databases and grey literature sources is conducted.
- Selecting studies: Pre-defined inclusion and exclusion criteria are applied.
- Extracting data: Pertinent information from the selected studies is collected.
- Collating, summarising, and reporting results: Narrative synthesis and data presentation are performed in tabular and graphical formats.

### 2.2. Ethical Considerations

This scoping review protocol does not involve the collection of primary data from human participants. Consequently, ethical approval from a Research Ethics Committee (REC) is not required. Nevertheless, all data will be extracted and synthesised in an ethical manner, with due regard for copyright regulations and established guidelines for scientific publication. The funding sources of the included studies will be identified and reported where such information is available.

### 2.3. Search Question

The central search question guiding this scoping review is

“What are the challenges faced by newly qualified nurses in leading nursing teams within hospital units?”

The search question was formulated using the PCC (Population, Concept, Context) framework:

- Population: The population included newly qualified nurses (defined as those with up to two years of post-graduation experience).
- Concept: The concept was challenges in leading the nursing team.
- Context: The context was hospital units.

### 2.4. Inclusion and Exclusion Criteria

To ensure that the scoping review remained focused on the most germane and informative studies, rigorous inclusion and exclusion criteria are applied throughout the article selection process. Studies that directly address the challenges experienced by newly qualified nurses in leading nursing teams are included. The context of these studies must be hospital units, reflecting the specific environment of interest. The definition of “newly qualified nurse” is standardised to encompass professionals with up to two years of post-graduation experience, thereby ensuring a homogenous sample. The review considers studies employing a range of methodological designs, including qualitative, quantitative, mixed methods, literature reviews, and case studies, to obtain a comprehensive understanding of the topic. Articles published in Portuguese, English, and Spanish are included to ensure broad coverage of the available literature. The selection of these three languages is based on their prevalence in the relevant nursing and health science literature and the research team’s linguistic capabilities, allowing for a comprehensive yet feasible review of the evidence base within these linguistic domains.

Conversely, studies that do not address the leadership challenges encountered by newly qualified nurses, as well as those conducted outside the hospital setting, are excluded. Studies that do not specifically focus on newly qualified nurses, or that do not meet the defined criterion of up to two years of experience, are also excluded. Opinion pieces, editorials, letters to the editor, and conference abstracts are excluded on account of their non-empirical nature.

### 2.5. Search Sources

The identification of relevant studies was accomplished through a comprehensive search of diverse information sources. The primary databases consulted include MEDLINE (via PubMed), CINAHL (via EBSCOhost), Web of Science, Scopus, LILACS (via Virtual Health Library), and SciELO. These databases are recognised for their extensive coverage of the scientific literature in the fields of health and nursing.

In addition to the aforementioned databases, a search of grey literature sources, such as theses and dissertations available in digital libraries and institutional repositories, was also undertaken. The inclusion of grey literature is important for identifying studies that may not have been published in peer-reviewed journals but may nevertheless provide valuable insights into the topic under investigation. The search strategy will be adapted to suit the specific characteristics of each information source, in order to maximise the likelihood of identifying all relevant studies.

### 2.6. Search Strategy

The search strategy will be developed in collaboration with a librarian possessing expertise in systematic reviews. The following search terms will be employed, in conjunction with Boolean operators (AND, OR): [Insert specific search terms and combinations here, e.g., “newly qualified nurse*” OR “new graduate nurse*” AND “leadership” OR “management” AND “challenge*” OR “barrier*” AND “hospital*” OR “inpatient unit*”].

### 2.7. Study Selection

The selection of studies was conducted in two phases:

- Title and Abstract Screening: Two independent reviewers (identified as Reviewer 1 and Reviewer 2) analysed the titles and abstracts of the articles identified during the search, applying the inclusion and exclusion criteria. Discrepancies were resolved by consensus or by a third reviewer (Reviewer 3). The Rayyan QCRI tool was utilised to facilitate the screening process of titles and abstracts. Rayyan was chosen for its efficiency in managing the screening process for multiple reviewers, facilitating blinding and conflict resolution.
- Full-Text Review: Articles selected in the first phase were read in full by two independent reviewers (Reviewer 1 and Reviewer 2), who again applied the inclusion and exclusion criteria. Discrepancies were resolved by consensus or by a third reviewer (Reviewer 3). Additionally, the reference lists of all studies included after the full-text review were screened for potentially relevant articles.

The study selection process was documented using a PRISMA-ScR flowchart, which includes the number of articles identified in each database, the number of articles eliminated due to duplication, the number of articles excluded in title and abstract screening, the number excluded in the full-text review, and the number of articles included in the review.

### 2.8. Data Extraction

Data will be extracted from the selected articles using a standardised form developed by the reviewers (Appendix A). Two independent reviewers (Reviewer 1 and Reviewer 2) extracted the data from the articles, and discrepancies were resolved by consensus or by a third reviewer (Appendix B). A pilot test of the data extraction form was conducted with a randomly selected group of articles to ensure its clarity and usability.

### 2.9. Data Analysis and Synthesis

The extracted data were analysed descriptively, employing simple statistics (frequencies, percentages) to summarise the characteristics of the studies and the main challenges identified. The results are presented in tables and charts, and a narrative synthesis is developed to describe the main themes and patterns identified in the literature.

### 2.10. Article Analysis Mechanism

To ensure consistency in the analysis of the articles, an analysis guide developed by the reviewers was used (Appendix B). The analysis instrument was tested on a randomly selected group of articles, and the reviewers discussed and resolved any doubts or inconsistencies before conducting the complete analysis of the articles.

### 2.11. Presentation of Results

The results of the scoping review are presented clearly and concisely, using tables, charts, and a narrative synthesis. The results presented include

- The number of articles identified in each database and within the grey literature;
- The number of articles included and excluded at each stage of the selection process;
- The characteristics of the included studies (study design, sample size, country of origin, etc.);
- The characteristics of the participants (profile of newly graduated nurses);
- The main challenges identified in nursing team leadership, categorised by theme;
- The strategies used to overcome these challenges, categorised by type;
- The results of the methodological quality assessment of the included studies;
- Knowledge gaps and recommendations for future research;
- The narrative synthesis, which describes the main themes and patterns identified in the literature and presents a critical analysis of the results.

## 3. Results

This section outlines how the findings of the completed scoping review are presented, with a specific emphasis on elucidating the unique challenges faced by newly qualified nurses exclusively within hospital units. The results of this scoping review are presented in both tabular and narrative formats. Tables provide a concise summary of the data extracted from the included studies, highlighting key study characteristics (such as design, sample, context) and the principal leadership challenges identified. The tabular presentation facilitates an easy and rapid comparison of results across different studies. Crucially, this tabular presentation is structured to facilitate a clear comparison and differentiation of these challenges as they specifically manifest in the hospital environment for this particular population, distinguishing them from broader nursing leadership issues.

The narrative synthesis complements the tables by offering a detailed and contextualised interpretation of the findings. This synthesis integrates both quantitative and qualitative discoveries, seeking to identify patterns, recurring themes, and knowledge gaps that are specific to the experience of newly qualified nurses in hospital units. Specifically, the narrative synthesis provides an in-depth explanation of the main challenges identified, detailing how they manifest uniquely in the hospital setting. This includes a detailed description of the types of conflicts encountered (e.g., interpersonal, intrapersonal, organisational), the nature of decision-making under duress, specific communication barriers, and the complexities of adapting to leadership role expectations, all framed within the distinct pressures and demands of the hospital environment. Furthermore, the narrative synthesis elaborates on the strategies reported in the literature to overcome these challenges, providing examples and context relevant to this specific context. Additionally, the narrative synthesis explores the relationships between the various leadership challenges and the contextual factors that may influence them, thereby highlighting the nuanced differences from general nursing leadership challenges.

Collectively, the tabular presentation and narrative synthesis provide a comprehensive and in-depth overview of the research landscape concerning the leadership challenges faced by newly graduated nurses in hospital units, directly addressing the primary research question and providing a clear understanding of the identified challenges and potential strategies. This focused approach distinctly highlights the specificities of these challenges within the hospital context, thereby differentiating our findings from the broader literature on nursing leadership and new graduate transition and contributing a granular understanding to the field.

## 4. Discussion

This scoping review protocol provides a comprehensive overview of the challenges newly graduated nurses face in leadership roles within hospital units. The findings highlight several key challenges, including conflict management, decision-making under pressure, and effective communication, which align with the existing literature on nursing leadership. These challenges underscore the complexity of transitioning from academic training to professional practice, as noted by Salem Alghamdi and Ghazi Baker [9].

The results indicate that newly qualified nurses often struggle with integrating theoretical knowledge into practical scenarios, a finding consistent with previous studies [12]. This difficulty is compounded by a lack of practical experience, which can lead to insecurity and hinder effective team management. Moreover, the necessity of fostering a collaborative environment is crucial for successful leadership, as emphasised by Huynh [14].

In terms of new knowledge gaps, this review identifies a need for targeted interventions that address these specific challenges. While mentorship programmes and training initiatives in communication and conflict management have been suggested as potential solutions [11], further research is needed to evaluate their effectiveness in diverse hospital settings.

The methodological transparency of this review ensures that the findings are robust and reproducible, providing a solid foundation for future research. However, the heterogeneity of the included studies presents a limitation, as it may affect the comparability of the results. Future studies should aim to standardise methodologies to enhance the reliability of findings.

In conclusion, this scoping review not only maps the challenges faced by newly graduated nurses, but also highlights the importance of developing comprehensive support systems to enhance their leadership capabilities. By addressing these challenges, healthcare institutions can improve both the quality of care and the professional satisfaction of nursing staff.

### 4.1. Expectations with the Protocol Development

It is expected that this detailed protocol will serve as a clear and comprehensive guide for conducting the scoping review, minimising the risk of bias and ensuring the quality of the results. We believe that the detailed description of the methods for searching, selecting, extracting, and analysing data will allow other researchers to replicate this study and assess the validity of its conclusions.

### 4.2. Importance of the Scoping Review

The scoping review is an appropriate methodology for this topic as it seeks to identify the extent and nature of the available evidence, rather than evaluating the quality or effect of interventions [23]. Identifying the challenges faced by newly graduated nurses in leadership is crucial for guiding future research and interventions aimed at improving healthcare quality and the professional satisfaction of these professionals.

### 4.3. Possible Limitations of the Protocol

While this study was conducted with methodological rigour, several limitations must be acknowledged. Firstly, the analysis is limited to publications in three languages: English, Portuguese, and Spanish. This may have excluded relevant studies published in other languages, potentially impacting the comprehensiveness of the review.

Additionally, the heterogeneity of the included studies, encompassing different methodological designs and samples, may hinder the comparison of results and the accomplishment of a comprehensive narrative synthesis. This diversity can introduce variability that affects the consistency of the findings.

Furthermore, the methodological quality assessment of the studies was conducted using different tools, which may introduce some subjectivity into the analysis. This could affect the reliability of the conclusions drawn.

Despite these limitations, this review provides valuable insights into the challenges faced by newly graduated nurses in leadership roles and highlights areas for future research.

### 4.4. Future Directions

Based on this protocol, it is anticipated that the scoping review will provide a comprehensive overview of the challenges faced by newly graduated nurses in leadership. The review results could be used to

- Identify knowledge gaps regarding the topic;
- Guide future research, investigating the factors influencing leadership development in nursing and the effectiveness of different interventions;
- Develop more effective support and training programmes for newly graduated nurses;
- Inform the formulation of nursing management policies and practices.

## 5. Conclusions

This scoping review has identified several key challenges faced by newly graduated nurses in leadership roles, including conflict management, decision-making under pressure, and effective communication. These challenges highlight the complexity of transitioning from academic training to professional practice and underscore the need for targeted interventions.

The practical implications of these findings suggest that healthcare institutions should develop comprehensive support systems, such as mentorship programmes and training initiatives, to enhance the leadership capabilities of newly graduated nurses. Such interventions are crucial for improving healthcare quality and ensuring professional satisfaction among nursing staff.

Future research should focus on evaluating the effectiveness of these interventions across diverse hospital settings and exploring additional strategies to support newly graduated nurses. By addressing these challenges, future studies can contribute to the development of more effective nursing management policies and practices.

In summary, this review provides a comprehensive overview of the challenges and offers valuable insights for guiding future research and practice. The findings emphasise the importance of supporting newly graduated nurses in their leadership roles to enhance both patient care and professional development.

## Data Availability

The original contributions presented in this study are included in the article. Further inquiries can be directed to the corresponding author.

## Public Involvement Statement

There was no public involvement in any aspect of this research.

## Guidelines and Standards Statement

This manuscript was drafted against the PRISMA-ScR (Preferred Reporting Items for Systematic reviews and Meta-Analyses extension for Scoping Reviews) guidelines (Tricco et al., 2018) [22] for scoping review research.

## Appendix A

**Table A1:** Data extraction instrument.

Item	Description
**General Study Information**	
Study Title	Full title of the article.
Authors	Complete list of authors.
Year of Publication	Year in which the article was published.
Country of Origin	Country where the research was conducted.
Language of Publication	Language in which the article was published.
Type of Study	Qualitative, Quantitative, Mixed, Literature Review, Case Study, etc.
Study Objective	What is the main objective of the research?
Research Question	What question does the research seek to answer?
Study Design	If applicable, detail the study design (e.g., Randomised Controlled Trial, Cohort Study, etc.).
Data Collection Period	When was the data collected?
Sources of Funding	Who funded the research?
Conflicts of Interest	Did the authors declare any conflicts of interest?
Ethical Considerations	Approval by Ethics Committee, Informed Consent, etc.
**Study Context**	
Type of Hospital	Public, Private, University, etc.
Hospital Size	Number of beds, Number of staff.
Hospital Location	Urban, Rural, etc.
Type of Unit	Medical Clinic, Surgical, Intensive Care Unit (ICU), etc.
**Sample Characteristics**	
Sample Size	Total number of participants.
Inclusion Criteria	What criteria were used to include participants in the study?
Exclusion Criteria	What criteria were used to exclude participants from the study?
Sampling Method	Probabilistic, Non-Probabilistic (e.g., Convenience, Simple Random, etc.).
**Profile of Newly Graduated Nurses**	
Gender	Number and percentage of males and females.
Age	Mean and standard deviation, or age range.
Experience Duration	Mean and standard deviation, or range (in months).
Educational Level	Undergraduate, Postgraduate, Specialisation, etc.
Other Characteristics	Specify (e.g., area of practice, type of contract, etc.).
**Data Collection Methods**	
Type of Data Collection	Primary, Secondary.
**Data Collection Instruments**	
Questionnaires	Scales used, Validity, and Reliability (if reported).
Interviews	Structured, Semi-Structured, Unstructured. Is an interview guide available?
Observation	Participant, Non-Participant. Is there a standardised observation instrument?
Document Analysis	Medical records, Reports, etc.
Other Methods	Specify.
Data Collection Procedures	How was the data collected? (e.g., individual interview, online questionnaire application, etc.)
Training of Data Collectors	Did the data collectors receive training? If so, what kind?
Data Quality Assurance	What measures were taken to ensure data quality? (e.g., double-checking, data validation, etc.)
**Data Analysis Methods**	
Type of Analysis	Qualitative, Quantitative, Mixed.
Statistical Analysis	Tests used, Level of significance.
Qualitative Analysis	Type of analysis (e.g., content analysis, thematic analysis, etc.), Software used (if applicable).
Validation of Results	Triangulation, Peer Validation, etc.
**Main Challenges Identified in Nursing Leadership**	
Challenges Related to Conflict Management	Types of Conflicts (Interpersonal, Intrapersonal, Organisational), Causes of Conflicts, Conflict Resolution Strategies. Cite specific examples found in the study.
Challenges Related to Decision-Making	Types of Decisions (Routine, Complex, Urgent), Factors Influencing Decision-Making. Cite specific examples found in the study.
Challenges Related to Effective Communication	Types of Communication (Verbal, Non-Verbal, Written), Barriers to Communication. Cite specific examples found in the study.
Challenges Related to Role Expectation Adaptation	Expectations of Colleagues, Expectations of Superiors, Expectations of Patients. Cite specific examples found in the study.
Challenges Related to Lack of Practical Experience	Difficulties in Applying Theoretical Knowledge, Insecurity in Performing Procedures. Cite specific examples found in the study.
Challenges Related to Leadership Skill Development	Difficulties in Task Delegation, Difficulties in Team Motivation. Cite specific examples found in the study.
Other Challenges	Specify and cite specific examples found in the study.
**Strategies Used to Overcome Challenges**	
Mentorship Programmes	Characteristics of Mentorship Programmes, Results of Mentorship Programmes.
Communication Skills Training	Types of Training, Training Results.
Conflict Management Training	Types of Training, Training Results.
Support from Experienced Leaders	Types of Support, Results of Support.
Other Strategies	Specify.
**Study Results and Conclusions**	
Main Results	Detail the quantitative and qualitative results. Include relevant numerical data (e.g., means, standard deviations, *p*-values).
Conclusions	Summary of the study’s main conclusions.
Implications for Practice	How can the results be applied in practice?
Recommendations for Future Research	Authors’ suggestions for future studies.
Limitations of the Study	Identify the study’s methodological limitations.
**Other Relevant Information**	
Additional Comments	Additional information not covered in previous categories.
Study Quality Assessment	Use a quality assessment scale (e.g., Newcastle-Ottawa Scale for observational studies, Cochrane Risk of Bias Tool for clinical trials). Indicate the total score and justify the assessment.
Extractor’s Notes	Extractor’s notes on the study (e.g., strengths, weaknesses, relevance to the review, etc.).

Source: Prepared by the authors.

## Appendix B

**Table A2:** Instrument for analysis of selected articles.

Item	Description
**Study Identification**	
Study Title	Full title of the article.
Authors	Complete list of authors.
Year of Publication	Year in which the article was published.
**Study Objective**	
Main Objective	What is the primary objective of the research?
Research Question	What question does the research seek to answer?
**Study Design**	
Type of Study	Qualitative, Quantitative, Mixed, Literature Review, Case Study, etc.
Study Design Description	Detailed description of the study design (e.g., Randomised Controlled Trial, Cohort Study, Cross-Sectional Study, Case–Control Study, Ethnographic Study, etc.).
**Sample**	
Sample Size	Total number of participants.
Sampling Method	Probabilistic, Non-Probabilistic (e.g., Convenience, Simple Random, Stratified, etc.).
Inclusion Criteria	What criteria were used to include participants in the study?
Exclusion Criteria	What criteria were used to exclude participants from the study?
**Data Collection**	
Data Collection Methods	Questionnaires, Interviews, Observation, Document Analysis, etc.
Data Collection Instruments	Scales used, interview guides, observation protocols, etc.
Validity and Reliability	Validity and reliability of data collection instruments (if reported).
Data Collection Procedures	How was the data collected? (e.g., individual interview, online questionnaire application, etc.)
**Data Analysis**	
Data Analysis Methods	Statistical Analysis, Content Analysis, Thematic Analysis, etc.
Statistical Tests	Statistical tests used (if applicable).
Software Used	Software used for data analysis (if applicable).
**Results**	
Main Results	Summary of the study’s main findings.
Statistical Significance	Statistical significance level of the results (if applicable).
Validity of the Results	Are the results valid and reliable? Justify.
**Discussion**	
Interpretation of Results	How do the authors interpret the results?
Implications for Practice	What are the implications of the results for practice?
Study Limitations	What are the limitations of the study?
**Methodological Quality**	
Risk of Bias	Assessment of bias risk (e.g., selection bias, information bias, measurement bias, etc.).
Internal Validity	How reliable are the study results?
External Validity	How generalizable are the study results?
**Relevance**	
Relevance to the Review	How relevant is the study to the research question of the review?
Contribution to Knowledge	What does the study contribute to the knowledge on the subject?
**Ethical Considerations**	
Ethics Committee Approval	

Source: Prepared by the authors.
